# Lithium solvation and anion-dominated domain structure in water-in-salt electrolytes

**DOI:** 10.1039/d5eb00105f

**Published:** 2025-08-06

**Authors:** Timothy S. Groves, Kieran J. Agg, Shurui Miao, Thomas F. Headen, Tristan G. A. Youngs, Gregory N. Smith, Susan Perkin, James E. Hallett

**Affiliations:** a Physical and Theoretical Chemistry Laboratory, Department of Chemistry, University of Oxford Oxford OX1 3QZ UK timothy.groves@chem.ox.ac.uk; b Disordered Materials Group, ISIS Neutron and Muon Source, Rutherford Appleton Laboratory Didcot OX11 0QX UK; c Department of Chemistry, School of Chemistry, Food and Pharmacy, University of Reading Reading RG6 6AD UK j.e.hallett@reading.ac.uk

## Abstract

Water-in-Salt (WiS) electrolytes are an emerging class of high concentration aqueous electrolytes with large electrochemical stability windows, making them attractive as green alternatives in next-generation electrochemical energy storage devices. Recent work has highlighted the existence of water-rich and anion-rich domains in WiS electrolytes, but the extent, morphology and importance of these domains are still disputed. Here, we present neutron total scattering measurements of the archetypal WiS, lithium bis(trifluoromethanesulfonyl)imide, and use empirical potential structure refinement to match the structure of a simulated system to the experimental data for two technologically relevant concentrations, revealing ion solvation, geometric isomerism and long-range structures in unprecedented detail. Our analysis of the modelled WiS electrolyte suggests that water domains are small and isolated and points to a system dominated by percolating, anion-rich domains that assemble through the association of hydrophobic regions, extending throughout the entire system. This structural insight places restrictions on feasible transport mechanisms in WiSs and, more generally, will aid in the understanding of the structure and behaviour of WiS electrolytes, with implications for the design and manufacture of WiS-containing devices.

## Abstract 2

Neutron total scattering is used to reveal new details of the nanostructure of concentrated lithium bis(trifluoromethanesulfonyl)imide, the archetypal water-in-salt electrolyte.

## Graphical abstract

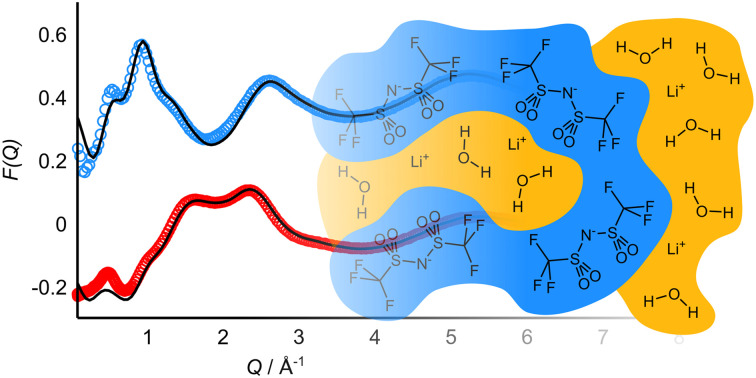

**Broader context**

Some of the earliest batteries were based on water, but their poor long-term stability and low energy density led to them being overtaken by organic electrolytes in rechargeable battery applications. The innovation of Water-in-Salt (WiS) electrolytes, where salt significantly outweighs water, displays greatly improved stability and energy density, alongside fast ion transport values, leading to a critical re-evaluation of purely aqueous battery chemistries. WiS electrolytes are thought to owe some of their impressive characteristics to the unique solvation and long-range structures present. Water-rich and anion-rich domains form, leading to more complex and diverse solvation environments than for a traditional battery electrolyte. However, both the structure of the electrolyte and its impact on subsequent electrochemical and transport properties are disputed. In this work, we adopted the “experiment-informed” simulation approach of neutron total scattering combined with empirical potential structure refinement to determine the nanostructure of concentrated lithium bis(trifluoromethanesulfonyl)imide solutions – the archetypal WiS electrolyte – in unprecedented detail. We showed that while anionic networks persist throughout the electrolyte, narrow water-rich domains become more sparse with an increase in salt concentration, and lithium ions are increasingly solvated by anionic domains – which in turn show morphological differences at the intra- and inter-molecular levels. This insight can lead to the optimisation of WiS solvation environments to further improve their electrochemical stability and transport properties.

## 1. Introduction

Lithium-ion batteries are essential to modern life, providing power in portable electronics and electric vehicles and allowing grid storage for renewable electricity. As the demand for electrochemical energy storage is set to grow in the coming years,^1^ increased attention has been given to the hazards that conventional lithium-ion batteries present. These can be at end-of-life, where the organic electrolytes are difficult to recycle and can cause environmental harm,^2–4^ and in use, where catastrophic battery failure can lead to fire hazards.^5^ Using water instead of conventional solvents can improve battery safety, yet the narrow electrochemical stability window (ESW) of aqueous electrolytes has limited device performance and lifetime, so water-based electrolytes have previously been overlooked as a viable alternative. However, in recent years Water-in-Salt (WiS) electrolytes have emerged as an aqueous alternative to traditional non-aqueous battery chemistries with comparable performance, reinvigorating interest in aqueous battery research.^6–8^

WiS electrolytes are extremely high-concentration aqueous salt solutions where the salt outnumbers the water both in terms of mass and volume fraction. Unlike the narrow ESW of dilute aqueous electrolytes, WiSs have a comparable ESW to conventional lithium-ion batteries,^8^ due to a combination of interfacial and bulk effects.^9^ At the interface, WiS electrolytes form a solid-electrolyte interphase (SEI)^10–12^ – a passivating film that protects the electrolyte from direct contact with the electrode while still allowing lithium ion transport, the structure of which is shaped by the bulk electrolyte.^13^ In the bulk, the solvation environment and nanostructure reduce water activity^13,14^ and disrupt the hydrogen bond network, disfavouring hydrogen evolution.^15^

This nanostructure also allows WiS electrolytes to show fast ion transport rates and high lithium ion transference numbers.^16,17^ Studies on the archetypal WiS, lithium bis(trifluoromethanesulfonyl)imide (LiNTf_2_, soluble in water up to 21 mol kg^−1^ at room temperature), have highlighted the existence of a nanosegregated structure with a characteristic length scale between 1 and 2 nm.^18–23^ In this picture, the WiS electrolyte is thought to be made up of two interpenetrating domains: a water rich domain and an anion rich domain, where the aqueous domain forms percolating channels in which a substantial portion of the lithium ions are solvated completely by water.^18,19,24^ These channels were proposed to act as a fast transport pathway for the hydrated lithium ions, with little change to the solvation shell of the ion as it travels *via* a vehicular mechanism. However, recent X-ray, FTIR and simulation studies^25–28^ have suggested that water exists only in small clusters and filaments at concentrations close to saturation. In this scenario a solvation hopping mechanism is thought to be responsible for rapid lithium ion transport,^26,28^ where the lithium solvation shell is exchanged over a molecular length scale and lithium is transported through both aqueous and anion domains. Alongside this ongoing discussion, there remain debates about the properties of WiSs, including around the dimensions and extent of water and anion domains, the relative diffusivities of each component, the degree of deprotonation and subsequent proton activity that occurs in the aqueous domains, and the relative amount of “bulk-like” or clustered water present.^24,26,29–32^

Solvation and nanoscale structure in the electrolyte play a key role in both the electrochemical stability and transport properties of WiS systems, and with the growing demand for green alternatives to conventional electrolytes, it is more pressing than ever to understand the structure and properties of WiS electrolytes at the nanoscale to inform the design of next-generation electrochemical energy storage. In order to disentangle the water structure and lithium solvation environment, here we report a neutron total scattering structural study of aqueous solutions of LiNTf_2_ within the WiS regime. We used Empirical Potential Structure Refinement (EPSR) to refine a simulation model against multiple scattering contrasts, revealing new details of the internal structure of the electrolyte. We found a 3D anion network surrounding extended – but not percolating – water domains. As the salt concentration is increased, the aqueous domains shrink in extent, supporting the idea that lithium transport cannot occur solely along water channels. The solvation structure becomes further perturbed from the dilute case with an increase in salt concentration, with highly distorted hydrogen bond networks in the aqueous domains and conformational changes to the NTf_2_ molecules in the anion domains. These findings provide new insights into the molecular signatures of WiS electrolytes that lead to their advantageous properties and will be instrumental in correlating molecular properties and liquid structures to the design of new electrochemical energy storage devices.

## 2. Experimental

Neutron scattering experiments were performed using the Near and InterMediate Range Order Diffractometer (NIMROD) at the ISIS Pulsed Neutron and Muon Source (Harwell, UK). The instrument has been described in detail elsewhere.^33^ Briefly, NIMROD utilises a broad range of neutron energies coupled with a detector array of ZnS scintillating detectors covering a solid angle from 0.5° to 40°, giving scattering information over a *Q* range of 0.02 Å^−1^ to 50 Å^−1^.

Samples were measured within the NIMROD instrument in titanium zirconium (TiZr) alloy cells at 298 K. Calibrations were taken with the empty instrument background, the empty cell backgrounds, and a vanadium niobium (VNb) alloy plate as a normalisation standard. Cells were then loaded with the sample at 298 K and exposed to the neutron beam for a minimum of 130 minutes. The measured neutron scattering data were reduced to the coherent elastic scattering contribution using the GudrunN program,^34^ in which the instrument and cell backgrounds were removed, and the data were normalised relative to the VNb plate. Inelastic scattering effects were subtracted using iterative methods developed by Soper.^35^ The measured differential cross section (DCS) was compared to the expected values for a sample matching the path length, density, and elemental composition of the prepared sample. Small variations in DCS from the expected values in D_2_O rich samples were attributed to excess H_2_O, likely from absorbed atmospheric water.

The normalised coherent scattering data were then fitted and analysed using the Dissolve software package (Dissolve v. 1.6.0),^36^ which works using the principles of EPSR.^37,38^ Briefly, a simulation box is constructed with the same molar ratios as the sample for which the scattering data are taken. Interactions within the system are initially described using a standard reference force field, then the system is evolved through a combination of Monte Carlo and molecular dynamics steps, and allowed to come to equilibrium as governed by the reference force field. Neutron scattering patterns are generated for the simulated box and compared to the measured scattering data. Additional empirical potentials can be introduced based on the differences between the measured and simulated scattering patterns and added to the force field to push the simulated data towards the measured scattering data. This process is repeated iteratively until a good match between the simulated and experimental scattering is found, at which point the distribution of species in the simulated box is taken as representative of the real sample. A trajectory of the box is recorded over 10 000 simulation frames at a frequency of every 5th frame, corresponding to 5 Monte Carlo steps and one molecular dynamics step.^36^ All data discussed in this article are extracted from these data points.

Samples of aqueous LiNTf_2_ were prepared from freshly opened bottles of LiNTf_2_ (Fluorochem, 99%). Solutions were prepared at four isotopic solvent contrasts: H_2_O, D_2_O, a 1 : 1 mixture of H_2_O and D_2_O known as HDO, and a 1.78 : 1 mixture of H_2_O and D_2_O for which the mean coherent scattering length of the water hydrogen atoms is zero, known as null water. Measurements were made at two concentrations for each contrast: 11.9 mol kg^−1^ and 19.7 mol kg^−1^. At 19.7 mol kg^−1^, we also prepared a sample using enriched ^7^LiNTf_2_ in D_2_O, following the method described by Maeda *et al*.^39^ The higher concentration is close to the room-temperature saturation limit of LiNTf_2_ (≈21 mol kg^−1^) with the widest ESW recorded,^6^ while the lower concentration allows comparison with structural forces observed in a recent surface force study^20^ (≈12 mol kg^−1^).

The simulation box at 11.9 mol kg^−1^ contained 6136 water molecules and 1317 LiNTf_2_ ion pairs (∼4.66 water molecules per ion pair) and was refined towards data collected in H_2_O, D_2_O, HDO, and null water. The simulation box at 19.7 mol kg^−1^ contained 5500 water molecules and 1953 LiNTf_2_ ion pairs (∼2.82 water molecules per ion pair) and was refined towards data collected in H_2_O, D_2_O, HDO, and null water with natural lithium, and data collected in D_2_O with ^7^Li. Initial force fields were taken from the literature.^40–43^ However, initial tests showed that on refinement, the added empirical potential could drive a small fraction of atoms to unphysical small separations. To prevent this, additional short-range repulsive potentials were added to some interactions. Details on the force field parameters and additional potentials are given in Tables S1–S4.

Measurements of densities of the H_2_O samples were made in order to calculate the number of atoms per cubic angstrom for the data reduction. Measurements were made using an oscillating U-tube density meter (Anton Paar, DMA 4100 M). At 11.9 mol kg^−1^ the density was found to be 1.61 g cm^−3^, or 0.0784 atoms per Å^3^. At 19.7 mol kg^−1^ the density was found to be 1.71 g cm^−3^, or 0.0745 atoms per Å^3^. A full concentration profile of the densities of aqueous LiNTf_2_ solutions was also made and is shown in Fig. S1.

Beyond analysis of the raw structural data, we performed a study of extended structures within the simulated trajectory. We used cluster analysis to determine the extent and make-up of extended structures using a method previously used to highlight water-rich regions present in deep eutectic solvents.^44^ Molecules are counted as being in the same cluster if defined atoms are within a certain distance of each other, with cut-offs chosen as the minima in the respective *g*(*r*)s. The probability *P*(*n*) of finding a cluster containing *n* molecules is compared to a theoretical cluster probability threshold^45,46^ to determine if the observed clusters are statistically significant. This method works to describe the species present in extended structures but gives no information on their shape. In order to characterise the shapes of any nanostructures, we performed a modified void analysis.^47^ Briefly, we deleted the atoms in a domain of interest from a trajectory of the simulated Dissolve box and then chose a random point *b* within the box and outside any remaining atoms. We then found the two atoms *a* and *c* nearest to this point. If the angle *abc* is close to 180°, then *a* and *c* lie on opposite sides of the volume left behind by deleting the atoms of interest, and thus the distance *ac* gives a lower estimate of the diameter of the domain of interest. Note that distances *ac* smaller than 2.4 Å are excluded from our analysis to prevent spurious contributions from outside the domain of interest. Diagrams illustrating the methodology behind these cluster and void analyses are shown in Fig. S2 and S3.

## 3. Results and discussion

### 3.1. Experimental fits

**Fig. 1:**
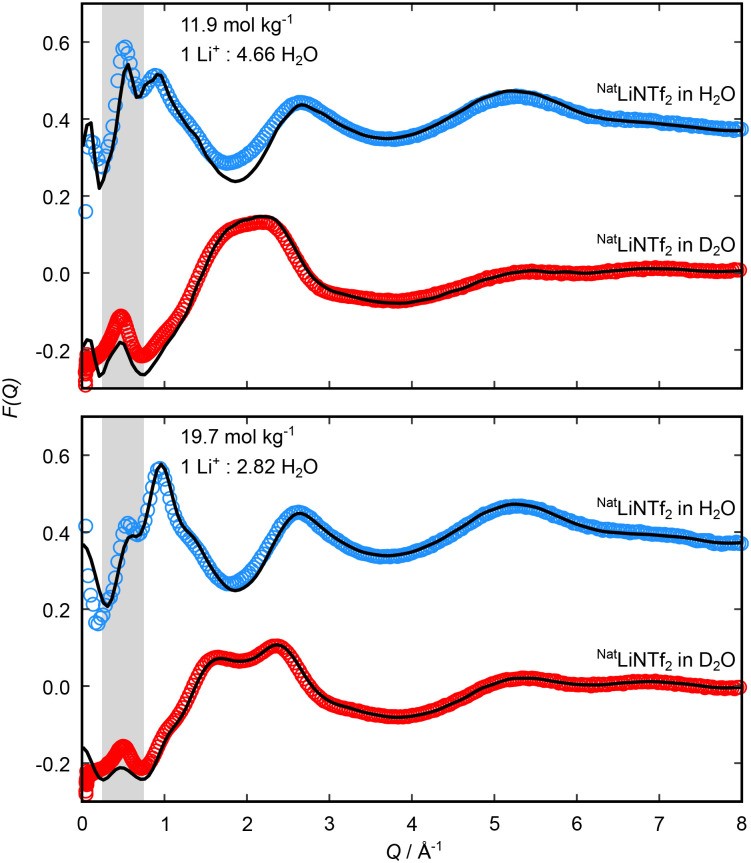
Total structure factors, *F*(*Q*), for aqueous solutions of ^Nat^LiNTf_2_ in H_2_O and D_2_O at 11.9 (top) and 19.7 mol kg^−1^ (bottom). The H_2_O data sets are shifted upwards for clarity. Experimentally measured data are shown as open symbols and the Dissolve model fit data are shown by a solid black line. The grey region highlights the peak in the low *Q* region. *F*(*Q*) measured and modelled data over the entire *Q* range and for additional isotopic contrasts are shown in Fig. S4 and S5.

Comparisons of the measured and fitted total structure factors, *F*(*Q*) for aqueous solutions of LiNTf_2_ in H_2_O and D_2_O at 11.9 and 19.7 mol kg^−1^ are shown in Fig. 1. For each contrast and at each concentration there is a clear prepeak both in the measured and fitted data at low *Q* centred at ≈0.5 Å^−1^, highlighted by the grey region in Fig. 1. Additional isotopic contrasts over a broader *Q* range alongside total pair distribution functions are shown in Fig. S4 and S5, while comparisons to previous experimental and simulation work are shown in Fig. S6 and S7.

In general, there is excellent agreement between the measured and fitted data, except at very low *Q* (<0.2 Å^−1^), where finite size effects in the simulation box can lead to a poor fit. Whilst the fitted data qualitatively matches the experimental data for all *Q* > 0.2 Å^−1^, there is also a slight baseline mismatch at 0.2 < *Q* < 1.0 Å^−1^, which likely arises from errors in the inelasticity correction. This is most pronounced in the D_2_O solvent contrast, which suggests some level of H_2_O contamination. The issue of light hydrogen complicating the inelastic scattering background is a well-known problem in neutron scattering measurements of this type.^35^ However, the fitted scattering is obtained from a best fit across all contrasts, so any discrepancies in some of the contrasts should not significantly affect our interpretation.

**Fig. 2:**
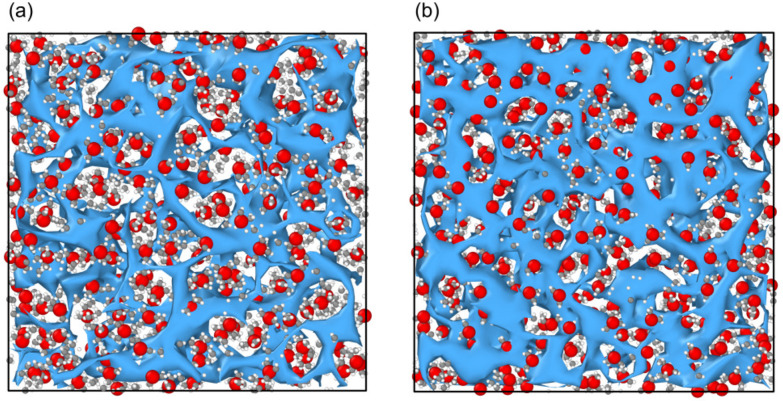
Snapshots of a 1.5 nm thick slice of the simulation frame at 11.9 mol kg^−1^ (a) and 19.7 mol kg^−1^ (b). Water oxygens are shown in grey, water hydrogens in white, lithium cations in red, and NTf_2_ anions as a blue mesh.

The low *Q* peak at ≈0.5 Å^−1^ corresponds to a real space correlation length *d* (*Q* = 2π/*d*) of 1.26 nm. This structural correlation is larger than any molecular length scale or nearest neighbour distance, so it can be attributed to the nanoscale structure in the liquid. Fig. 2 shows typical simulation frames for both sample compositions, with anions shown as a mesh. This representation highlights the nanostructural heterogeneity from fluctuations in anion and water density. This is in good agreement with previous scattering and surface force measurements, which have also seen long-range correlation lengths in similar concentration LiNTf_2_ solutions.^18,20,22^ By explicitly co-refining our simulation against multiple scattering contrasts, we were able to identify the structural origin of this length scale with greater specificity than previous studies. Previous work relied on qualitative comparison between simulated scattering and experimental data^17,18,23,25,48^ (see Fig. S6 and S7) to interpret the WiS structure but did not use the experimental data to refine the simulations. The use of multiple solvent isotopic substitutions also highlights the contribution of water domains to the total scattering intensity compared to experiments that rely on a single scattering contrast. This long-range structure, alongside coordination and intramolecular information (obtained at larger *Q*) is explored below.

### 3.2. Solvation and nanostructure

**Fig. 3:**
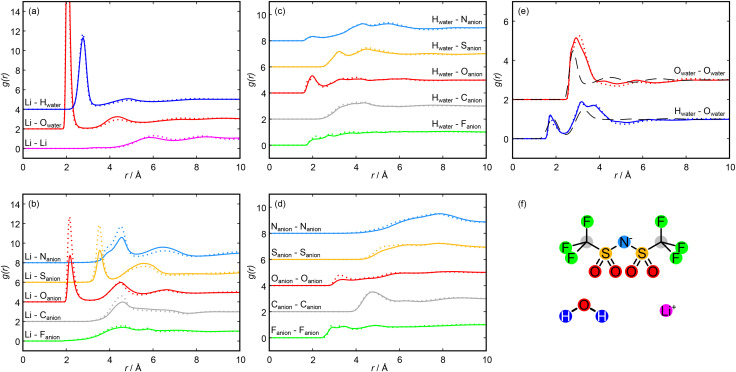
Intermolecular radial distribution functions (*g*(*r*)s) taken from the Dissolve model at 11.9 mol kg^−1^ (solid lines) and 19.7 mol kg^−1^ (dotted lines). *g*(*r*)s are accumulated over 10 000 frames. In each panel, different *g*(*r*)s are offset by 2 to aid clarity. (a) Lithium cation *g*(*r*)s with other lithium cations and with water molecules. (b) Lithium cation *g*(*r*)s with each atom of the NTf_2_ anions. (c) Water hydrogen atom *g*(*r*)s with each atom of the NTf_2_ anions. (d) NTf_2_ anion atom self *g*(*r*)s. (e) Water oxygen self *g*(*r*) and water oxygen–water hydrogen *g*(*r*) calculated from this work, compared with *g*(*r*)s for pure water reproduced from a work by Soper *et al.*^35^ (f) Molecular species present in this work. Each *g*(*r*) displayed in panels (a) to (e) is coloured according to this diagram.

**Fig. 4:**
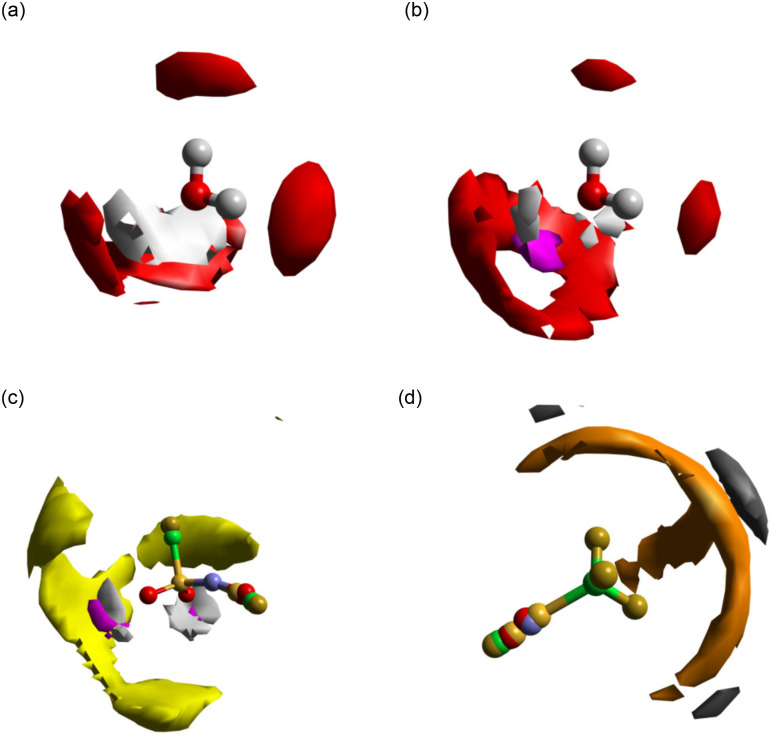
Spatial Density Functions (SDFs) showing the probability of coordination in the first solvation shell around molecules and groups. (a) Coordination of water hydrogen (white, 40% surface) and oxygen (red, 15% surface) around a water molecule in pure water (calculation made based on data reported by Soper *et al.*^35^). (b) Coordination of water hydrogen (white, 40% surface), water oxygen (red, 15% surface), and lithium (magenta, 36% surface) around a water molecule at 19.7 mol kg^−1^ LiNTf_2_. (c) Coordination of NTf_2_ sulfur (yellow, 15% surface), lithium (magenta, 10% surface) and water hydrogen (gray, 30% surface) around the anion SO_2_ group at 19.7 mol kg^−1^ LiNTf_2_. (d) Coordination of NTf_2_ fluorine (orange, 10% surface) and NTf_2_ carbon (black, 2.5% surface) around the anion CF_3_ group at 19.7 mol kg^−1^ LiNTf_2_. For clarity, only SDFs calculated from Dissolve simulations at 19.7 mol kg^−1^ LiNTf_2_ are displayed here. In all cases, analogous SDFs can be made for the system at 11.9 mol kg^−1^, and are displayed in Fig. S9. Note that the NTf_2_ molecular ions displayed in (c) and (d) are rotationally averaged about the respective functional groups that define the SDFs.

The nanostructuring prepeak captured experimentally (shown in Fig. 1) clearly decreases as the concentration is increased from 11.9 mol kg^−1^ to 19.7 mol kg^−1^. This decrease is well captured by the Dissolve model, as seen by the black simulated *F*(*Q*) in Fig. 1 as well as visually in the model snapshots in Fig. 2, for which we observed a decrease in water rich regions relative to anion rich regions: as the amount of salt is increased relative to the amount of water in the system, the water rich regions within the liquid will shrink. We can interpret the molecular configuration at the local level by considering the simulated radial distribution functions (*g*(*r*)s) and spatial density functions (SDFs) for each species. Fig. 3 shows *g*(*r*)s and Fig. 4 shows SDFs extracted from the Dissolve model.

We first turn to the solvation of the lithium cation, with *g*(*r*)s shown in Fig. 3(a) and (b). There is a very strong short-range correlation between the lithium and water oxygen atom, with an initial peak between 2.0 and 2.1 Å and a second, weaker peak at 4.4 Å at both concentrations. The lithium–water hydrogen *g*(*r*) shows similar features at both concentrations, with peaks shifted to slightly greater distances. This is consistent with an ion-dipole association with the cation, with the water oxygen oriented towards the lithium and extending to two hydration shells. For both the hydrogen and oxygen *g*(*r*)s, the second peak is weaker for the higher salt concentration, which suggests a second hydration shell is less likely as the salt concentration is increased. The presence of this favourable lithium association is enough to strongly disrupt the primary hydration shell of the water molecules, as can be seen in the SDFs shown in Fig. 4(a) and (b). The effect of this disruption will be discussed below.

The lithium–lithium *g*(*r*) shows a small prepeak at 3.5 Å followed by small peaks at 5.8 Å and 8.5 Å at 11.9 mol kg^−1^, consistent with previous simulation studies on lithium solvation.^49^ At the higher concentration, these small peaks shift slightly outwards while the prepeak is maintained. The prepeak likely corresponds to multiple lithium ions coordinated to the same water molecule or anion, with the later peaks arising from molecules in the second and third hydration shells of a reference lithium ion solvating a second lithium ion.

The lithium–O_anion_*g*(*r*) also indicates a strong interaction, as shown in Fig. 3(b). Here we observed three clear peaks: an initial peak at 2.2 Å, a second peak at 4.5 Å, and a third peak at 6.6 Å. These features are carried through the molecular ion, with clear peaks in the sulfur, nitrogen, carbon and fluorine–lithium *g*(*r*)s. These peaks do not speak of specific interactions but merely arise due to the interaction of lithium with the NTf_2_ oxygen atoms, with their distances reflecting the NTf_2_ ion geometry. In each case, the initial peak is much larger at the higher concentration, while the peaks at larger distances are comparable between the concentrations. The anion negative charge is spread across the oxygen atoms and the nitrogen atom, so these sites are able to interact electrostatically with the lithium cation; however, we observed no direct lithium–nitrogen interaction at a comparable distance to the lithium–oxygen interaction. This is likely because the nitrogen atom of the anion is sterically hindered by the rest of the anion, preventing a direct interaction with the lithium. The association of the lithium with the anion oxygens can be seen in the SDF shown in Fig. 4(c).

To allow for numerical comparison, we calculated coordination numbers of the first solvation shell, *N*, which can be calculated as the integral of a spherical shell containing the first peak in the *g*(*r*)s multiplied by the bulk density. The calculated coordination numbers for the total lithium–oxygen interaction, and with the water and the anion oxygens individually, are shown in Table 1. Full lithium–oxygen coordination histograms are shown in Fig. S8. The number of water molecules solvating each lithium ion decreases as the salt concentration is increased; however, the total lithium–oxygen coordination number is independent of mole fraction, as the dehydration is compensated by an increase in lithium–anion solvation as the concentration is increased. This suggests that extensive lithium–water domains are not as likely at very high salt concentrations, as they get broken up by lithium–anion interactions, similar to results from previous simulation studies.^6,18,25^ Importantly, this means that the fraction of lithium ions solvated solely by water molecules is low at the high concentration (∼13% compared to ∼38% at the low concentration).

**Table 1:** Coordination numbers, *N*, of the primary hydration shell of lithium ions at 11.9 m and 19.7 m LiNTf_2_ aqueous solutions. A cut-off value of 3.1 Å was used in each case, taken as the minima from the Li–O *g*(*r*)s shown in Fig. 3(a) and (b)

Environment	11.9 mol kg^−1^	19.7 mol kg^−1^
Li–O_total_	4.55	4.49
Li–O_water_	3.45	2.44
Li–O_NTf_2__	1.10	2.05

The correlations between the NTf_2_ ion and water molecules are shown by the water hydrogen–anion *g*(*r*)s in Fig. 3(c). The primary association is between the anion oxygen atoms and the water hydrogen, with a peak at 2.0 Å. At 19.7 mol kg^−1^, this peak appears to split, with a small shoulder peak at 1.8 Å. The position of this peak suggests a hydrogen bonding interaction,^35^ and the close H–O association may be seen in Fig. 4(c). This interaction again carries through the molecule, evidenced by minor peaks in the H–S and H–N *g*(*r*)s. There is also a small peak in the H–N *g*(*r*) at 2.0 Å, suggesting that this is a possible hydrogen bonding site. This could also account for the small shoulder seen in the Li–N *g*(*r*) at ≈3.9 Å (seen in Fig. 3(b)) as a lithium ion bound to the anion nitrogen atom *via* a bridging water molecule; however, these peaks are very small, which suggests that such an association is unlikely, possibly due to steric hindrance at the nitrogen site. There is little change in the peak positions as the concentration is increased, and a coordination number calculation shows that at 11.9 m, *N* = 0.45 for the O_anion_–H, while at 19.7 m, *N* = 0.50 (using a cut-off radius of 2.6 Å, taken as the minimum in the H–O *g*(*r*) shown in Fig. 3(c)). This suggests that as the concentration of salt is increased, the solvation environment of the water by the anion does not change significantly. This could mean that additional anions are dissolved into the anion rich domain only so that the region at the boundary between domains, where anion–water associations take place, is largely unchanged. Hence, this observation provides further evidence for the existence of a separation of the liquid into anion rich domains and water rich domains, with anion domains growing and water domains shrinking as the concentration is increased.

The *g*(*r*)s between like anion atoms are shown in Fig. 3(d). There is a pair of F–F peaks at 2.9 Å and 3.3 Å, a C–C peak at 4.7 Å, and a small O–O peak at 3.3 Å that becomes more prominent as the concentration is increased. Fig. 4(d) shows the associations between fluorine atoms and between carbon atoms. Fluorine atom density extends in a staggered manner about the CF_3_ group, with primary carbon atom probability density centred on the S–C bond axis and lower density in the plane of the CF_3_ fluorine atoms. This association likely arises because heavily fluorinated alkyl groups are hydrophobic and will associate with one another rather than with aqueous regions and has previously been seen in simulation studies,^18,25^ with the staggered arrangement allowing for a closer association. Overall, this means that the CF_3_ groups of the anion are pointing towards other anions, with the anion domain structure dominated by end-to-end associations, with a smaller contribution from side-to-side associations, and the increasing peak height with concentration suggests that these associations become more common at higher concentrations. Similarly, an intermolecular O–O association also becomes more prevalent at higher concentrations. This likely arises as the decrease in water concentration means that lithium ions and remaining water molecules are required to bind to multiple anions and is consistent with a shrinking of the aqueous domain. This association can be seen in the SDF shown in Fig. 4(c), with the S–O_water_ and S–Li association occurring in an initial shell about the anion oxygen atoms, with an outer shell showing the S–S association.

**Fig. 5:**
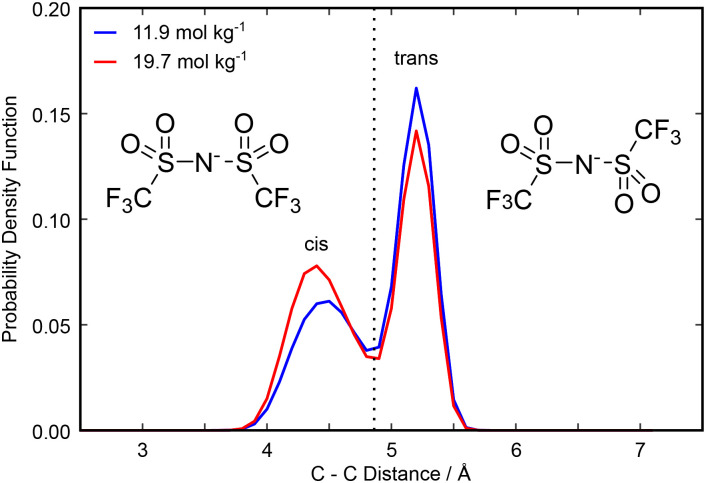
Probability density functions for the carbon–carbon distance in NTf_2_ ions in the Dissolve simulated box. The distribution found at 11.9 mol kg^−1^ is shown in blue and that found at 19.7 mol kg^−1^ is shown in red. The *cis* configuration is shown on the left and corresponds to a shorter C–C distance, while the *trans* isomer is shown on the right and corresponds to a longer C–C distance.

Considering now intramolecular associations of the anion, NTf_2_ may adopt either a *cis* or *trans* orientation with a low energy barrier to interconversion and the *trans* conformer representing the global minimum as there is less steric strain.^50,51^ The proportion of anions in our simulated box in either conformation can be determined by studying the intramolecular C–C distance. The two anion conformations and the probability distribution of the C–C distances at each concentration are shown in Fig. 5. We observed a bimodal distribution at each concentration, with a peak at 4.4 Å corresponding to a short C–C distance (the *cis* isomer) and a peak at 5.2 Å corresponding to a long C–C distance (the *trans* isomer). The *trans* isomer dominates the distribution at each concentration in our simulated box, as expected by steric constraints, with the *cis* isomer becoming more common as the concentration of salt is increased. The *trans* isomer has the SO_2_ hydrogen bond accepting oxygens on different sides of the molecule. As a result, it is able to act as a bridge between water-rich regions of the liquid and donate hydrogen bonds to multiple water molecules. Conversely, the *cis* isomer has all hydrogen bond accepting oxygen atoms on the same side of the molecule. This could create an environment in which it is difficult to hydrogen bond to multiple water molecules but allows for easy multidentate binding to lithium ions. Hence, the *cis* isomer becomes less disfavoured as the concentration of salt increases and the availability of water decreases, contributing to the changing domain structure of the liquid. Interestingly, for lithium–NTf_2_ in the absence of water, the most stable clusters prioritise the *trans* isomer,^52^ so one might expect an increased tendency for *trans* isomers within the NTf_2_ domains as the extent of and the amount of lithium within the domains increases, but instead we observed the opposite. Indeed, this effect is magnified when we consider only NTf_2_ ions that coordinate lithium (Fig. S10), which have a stronger tendency for the *cis* isomer than those that do not coordinate lithium, so it is likely that the shift in isomer ratio is related to the increase in the amount of lithium in the domain, rather than simply the extent of the domain increasing. We can attribute this to a conflict between the optimal network structure (taking into account interactions between the water- and anion-rich domains) and optimised solvation of the lithium ions. It might be that the most stable clusters for lithium–NTf_2_ in the WiS environment favour the *cis* isomer because they produce less unfavourable interactions with the aqueous phase than the *trans* isomer.

Fig. 3(e) shows the O_water_–H_water_ (blue) and the O_water_–O_water_ (red) *g*(*r*)s calculated in this work compared to equivalent *g*(*r*)s calculated from neutron scattering data in pure water,^35^ reproduced from a work by Soper *et al*. Similarly, Fig. 4(a) shows an SDF of pure water, calculated using data from Soper *et al.*, while Fig. 4(b) shows an SDF of water present in our concentrated system. The pure water data shows two O–H peaks at 1.9 Å and 3.3 Å, and three O–O peaks at 2.8 Å, 4.5 Å, and 6.8 Å. This distribution of peaks arises due to the extensive hydrogen bonding network that exists in pure water, with the hydration structure clearly defined out to three hydration shells. In the WiS electrolyte, however, we observed O–H peaks at 1.7 Å and 3.2 Å, similar to the pure O–H *g*(*r*), but we also observed an O–H peak at 3.8 Å, which is not present in the pure case. For the O–O *g*(*r*), we observed a single clear and broad O–O peak at 3.1 Å at 11.9 mol kg^−1^ moving to 2.9 Å at 19.7 mol kg^−1^ with a shoulder at 2.7 Å, with two small O–O peaks at 4.2 Å and 5.7 Å that become less well defined at higher concentrations. Coincident peaks suggest that some water–water hydrogen bonding is maintained in the WiS electrolyte, but the differences arise from the significant disruption to the pure water structure on the introduction of a large fraction of salt. The occurrence of a third O–H peak suggests an additional mode of water association that likely arises between water molecules that solvate a common lithium ion. This is also suggested in the O–O *g*(*r*), where the initial shoulder suggests hydrogen bonding, but the main peak is shifted to a greater distance, which can be accounted for by considering water oxygen atoms with interactions mediated by a lithium ion “bridge” rather than a hydrogen bond. The impact of the O–Li–O bridge can be seen in the SDFs presented in Fig. 4(a) and (b). In the case of pure water, the oxygen probability surface consists of two lobes close to the water hydrogens, arising from the H-bond network, and a large band above the water oxygen. In the case of the 19.7 mol kg^−1^ WiS electrolyte, this band becomes a ring around a region of lithium density – oxygen positions arising from hydrogen bonding between water molecules are maintained, but additional oxygen positions bridged to the water molecule by lithium ions are now apparent, which are less orientationally restricted than water molecules interacting *via* hydrogen bonds only. The distortion to the outer peaks in the O–O *g*(*r*) suggests that further hydrogen bond networks break down, with extended water structures unlikely in the 11.9 mol kg^−1^ WiS electrolyte and even less likely as the concentration is increased. The changes to native water structure are mirrored in the correlations between the NTf_2_ oxygens and water hydrogens, shown in Fig. 3(c). The shoulder at 1.8 Å for 19.7 mol kg^−1^ corresponds to water molecules more closely coordinating NTf_2_ oxygens, at the expense of forming a compact water hydrogen bond network, which is no longer optimal in the confined nanostructure.

From the O–O *g*(*r*), two important distances can be identified: the primary minimum in pure water is at 3.4 Å, corresponding to the hydrogen bonding interaction, and in the WiS electrolytes is at 4.0 Å, arising as a result of water molecules bridged by lithium atoms. Coordination numbers calculated using these cut-offs are shown in Table 2, and running coordination numbers are shown in Fig. S11. Again, we observed clear disruption to the primary hydration shell of water from the pure water case, with coordination as a result of hydrogen bonding falling from 4.7 water molecules in pure water to less than 3 water molecules in each of the aqueous electrolytes at the 3.4 Å cutoff, reflecting the low water concentration. The disruption is increased at the higher concentration, with the water coordination numbers falling further at both the H-bonding cutoff and the lithium bridging cutoff. This is in keeping with the experimental results given in Fig. 1, which show a decrease in the correlations from extended water domains as the salt concentration is increased. However, the data in Fig. 1 also shows that the length scale of these domains is constant at the two concentrations. This was also shown in a recent surface forces study,^20^ which showed a constant structural length scale in aqueous LiNTf_2_ solutions from 4.5 to 11.9 mol kg^−1^. Together, these results suggest that while extended aqueous domains become less likely as the salt concentration is increased, the correlation length scale between domains is unchanged. This could be as a result of narrowing water channels, in which water molecules form increasingly confined, chain-like structures as the concentration is increased; or this could be a sign of a break-up in the water domains into smaller, more isolated water-rich regions. To discriminate between these two cases, an approach that identifies the extent of domains is required, which we will describe in the following section.

**Table 2:** Coordination numbers, *N*, of the primary O_water_–O_water_ association in pure water,^35^ and at 11.9 m and 19.7 m LiNTf_2_ WiS solutions as measured in this work. Cut-off values of 3.4 Å and 4.0 Å (O_water_–O_water_ primary minima in measured *g*(*r*)) were used in each case, taken as the primary minima from the O_water_–O_water_*g*(*r*)s from the pure water and the WiS solutions shown in Fig. 3(e)

Cut-off/Å	11.9 mol kg^−1^	19.7 mol kg^−1^	Pure water
3.4	2.91	2.15	4.73
4.0	4.53	3.30	7.76

More broadly, our results for the solvation environments of different atomic and molecular species follow *general* trends reported in previous simulation work;^17,18,25,27,30^ however, we note several key differences that are highlighted by the neutron total scattering and EPSR method. We observed slightly stronger anion O–Li coordination here than typically reported,^17,18,25,27^ perhaps reflecting a higher degree of lithium solvation by the NTf_2_ domains (Fig. 3(b)) as the concentration is increased. This is also seen in the anion–water associations, which are weaker here than seen by Sha *et al*.^27^ Conversely, we observed weaker associations between carbon atoms in NTf_2_ ions than Zhang *et al*.,^25^ perhaps due to greater steric hindrance by incorporation of water or lithium into the NTf_2_ domains. We also observed differences in our water O–O *g*(*r*): we have discussed above our observation of a broad peak that shifts to larger distances away from the pure water initial solvation peak, which we attributed to the growing contribution from O–Li–O water bridges. This is similar to the observations of Yu *et al*.^17^ but differs from those of Zhang *et al*.,^25^ where the O–H–O peak continues to dominate at high concentration, and could highlight the decreasing role of bulk-like water molecules seen here. Finally, we observed no strong lithium–lithium association at either studied concentration, in contrast to previous simulation work in this electrolyte that reported the formation of polymer-like lithium–water nanochains.^21^ We propose that these differences are due to a greater contribution of the NTf_2_ to lithium solvation *in the water channels*, acting to distort their hydration structures. This behaviour at the interface of the water-rich and anion-rich domains is captured by our combined experiment-simulation approach, but not by previous simulations alone.

### 3.3. Network formation

**Fig. 6:**
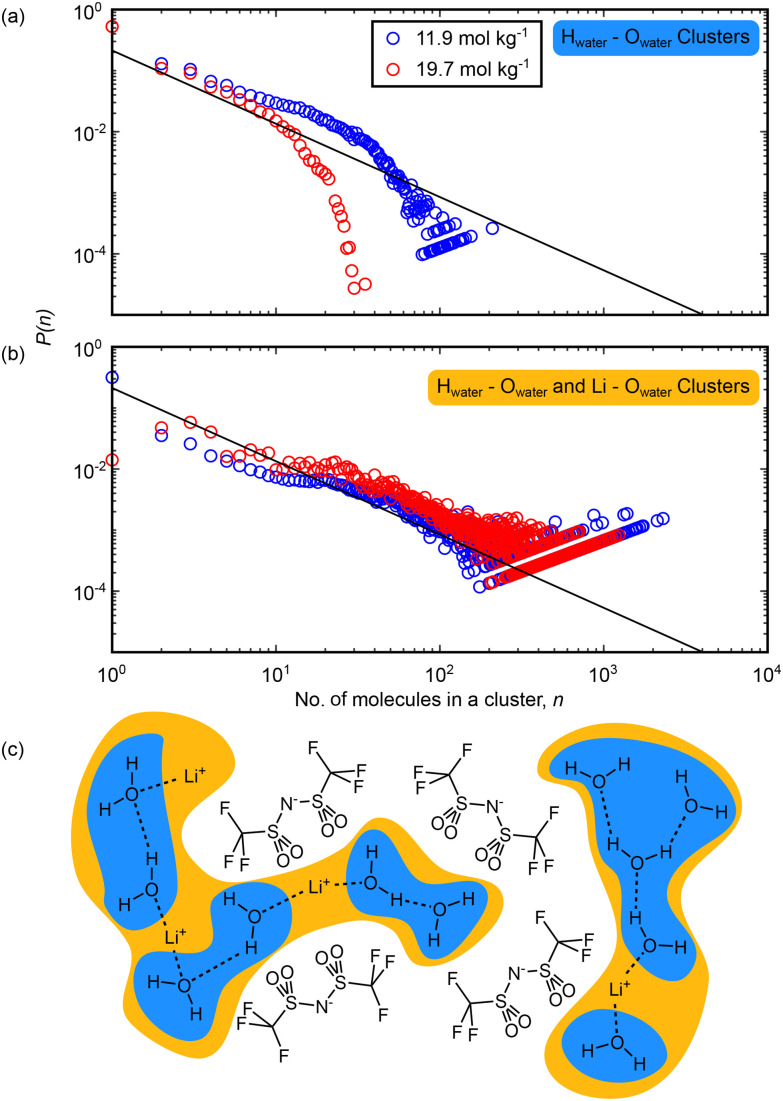
Cluster analysis of the simulation boxes in aqueous LiNTf_2_ showing the extent of domains mediated by interactions between water molecules. Clusters are counted every 50 frames over a trajectory of 10 000 frames. (a) and (b) Probability *p*(*n*) of finding a molecule within a cluster containing *n* molecules against the size of the cluster, *n*. Clusters counted at 11.9 mol kg^−1^ LiNTf_2_ are shown in blue and those counted at 19.7 mol kg^−1^ are shown in red. The solid black lines on the plots show the percolation threshold normalised to the box size.^45,46^ (c) Cartoon depiction of the cluster definitions used above: in (a), blue clusters focusing on only O_water_–H_water_ distances <2.3 Å, while in (b), yellow clusters including both O_water_–H_water_ and O_water_–Li^+^ distances <2.9 Å are shown.

We now turn to the results of the cluster analysis, first considering the make-up of the water-rich domains. The cluster analyses are shown in Fig. 6. We considered two interactions: we looked at clusters formed only by water–water hydrogen bonds, defined by the O–H distance, and second, we included water molecules bridged by lithium ions, defined by the O–Li distance. We found that at 11.9 mol kg^−1^, extended regions of water–water bridges (Fig. 6(a)) are probable up to clusters containing ∼50 molecules. At 19.7 mol kg^−1^, this is reduced and clusters with more than ∼10 molecules are unlikely. This is consistent with our previous analysis: extended water-rich regions decrease in size as the concentration is increased. Broadening this to include water–lithium interactions (Fig. 6(b)), we observed that extended domains of water and lithium now exist and are statistically significant up to ∼2000 molecules at 11.9 mol kg^−1^ and up to ∼1000 molecules at 19.7 mol kg^−1^. This highlights the important role of lithium in bridging between regions of water density. However, it is important to note that at neither concentration does the size of the largest detected cluster approach the largest possible cluster (7453 molecules and ions, containing all water molecules and lithium ions in the box). This suggests that at concentrations of 11.9 mol kg^−1^ and above, there is never a fully percolating network of water-rich domains. Thus, transport of lithium ions through the WiS electrolyte cannot take place solely through aqueous domains, and some diffusion through the anion rich domain will be needed, either as lithium ions solvated directly by anions or as diffusion of the total aqueous cation domain structure.

**Fig. 7:**
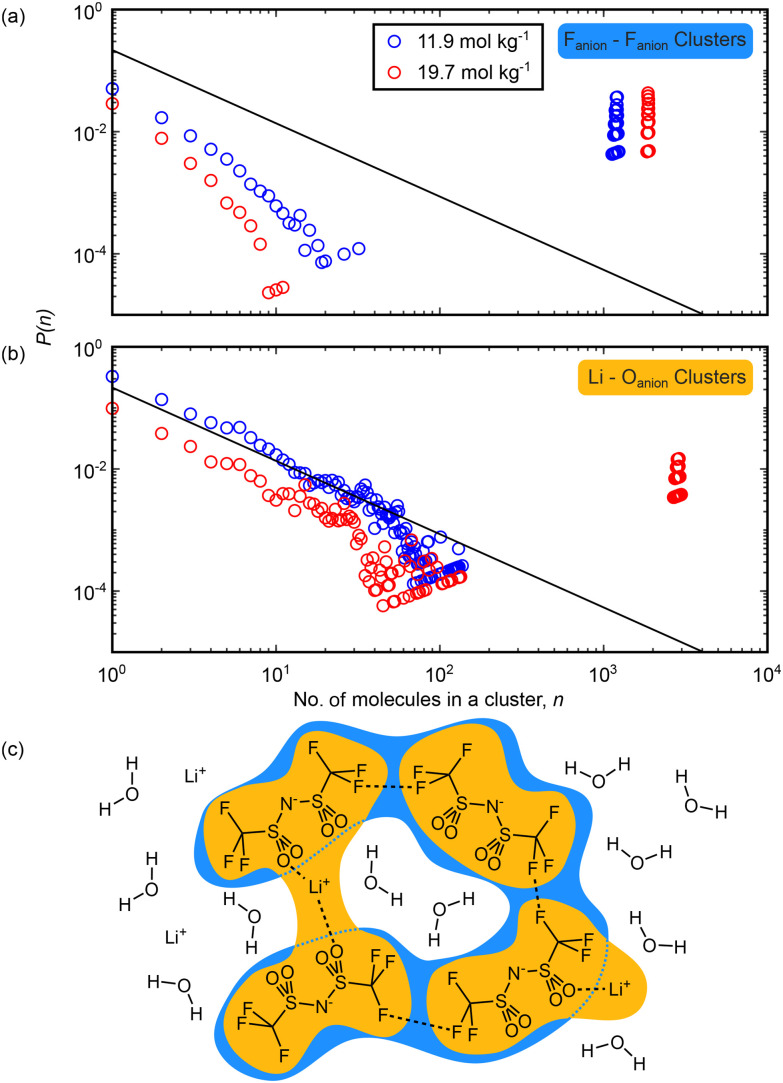
Cluster analysis of the simulation boxes in aqueous LiNTf_2_ showing the extent of domains mediated by interactions between anions. Clusters are counted every 50 frames over a trajectory of 10 000 frames. (a) and (b) Probability *p*(*n*) of finding a molecule within a cluster containing *n* molecules against the size of the cluster, *n*. Clusters counted at 11.9 mol kg^−1^ LiNTf_2_ are shown in blue and those counted at 19.7 mol kg^−1^ are shown in red. The solid black lines on the plots show the percolation threshold normalised to the box size.^45,46^ (c) Cartoon depiction of the cluster definitions used above: in (a), blue clusters focusing on only F_anion_–F_anion_ distances <3.1 Å, while in (b), yellow clusters focusing on only F_anion_–Li^+^ distances <3.1 Å are shown.

We also used this approach to consider the three-dimensional structure of the anion-dominated domain, with the cluster analyses shown in Fig. 7. We considered clusters comprised solely of NTf_2_ ions, defined by the F–F closest approach distance, and clusters of NTf_2_ bridged by lithium ions, defined by the O–Li distance, at both concentrations. When looking at the intermolecular F–F distance (Fig. 7(a)) we found evidence at each concentration for an extended anion network that contains close to all the anions in the simulation box. The formation of this network is likely driven by hydrophobic interactions between highly fluorinated anion CF_3_ groups, as has previously been found in simulation and small angle studies,^22^ and the presence at both concentrations of this network in our study highlights the scale of the segregation into anion-rich and water-rich domains. Focusing instead on the anion–lithium interactions by performing a cluster analysis defined by the O–Li distance (Fig. 7(b)), we observed that extended domains are more likely than chance only to groups of about 10 ions at 11.9 m. This suggests that at the lower concentration studied, lithium cations and NTf_2_ anions are not available to bind to each other in large groups, and likely there is sufficient water present to solvate each ion and prevent large regions of cation–anion dominated liquid structure. As the concentration was increased to 19.7 m, we did observe the emergence of an extended ion-only network. This implies that as the concentration is increased, ions become free to directly interact with each other as they lose water solvation. This can lead to the observed ion-dominated network as lithium ions are now able to act as bridges between anions across the aqueous domains. Further to this, lithium will also begin to dissolve in the anion rich domains as the water-rich domains shrink. This can be seen from the change in lithium coordination number histograms shown in Fig. S8.

**Fig. 8:**
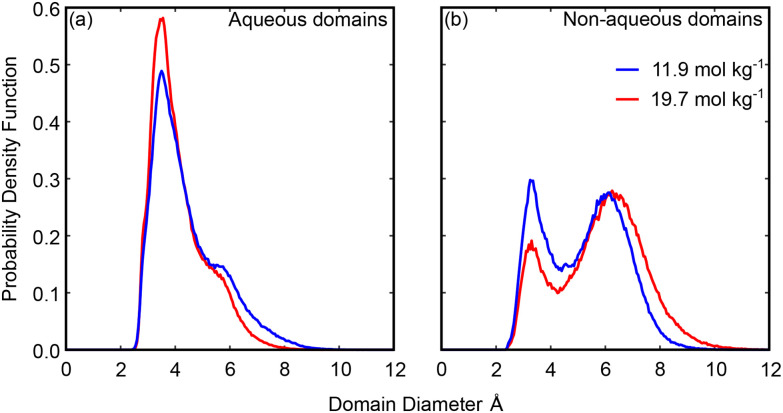
Probability density functions for the diameters of the domains present in concentrated aqueous LiNTf_2_. Domains found at 11.9 mol kg^−1^ are shown in blue and those found at 19.7 mol kg^−1^ are shown in red. (a) Diameters of voids left behind on deleting water molecules and lithium ions, *i.e.* a measurement of the aqueous domains. (b) Diameters of voids left behind on deleting NTf_2_ molecular ions and lithium ions, *i.e.* a measurement of the non-aqueous domains.

Turning now to study the shapes of these domains, we looked at the results of the modified void analysis described in the methods section to determine the smallest radial dimension of these domains. We studied the anion-rich domain by deleting all anion and lithium atoms and the water-rich domain by deleting all water and lithium atoms. The probability density functions showing the distribution in domain diameters are shown in Fig. 8. It is important to remove the lithium ions as they will be present within each domain, as shown by the cluster analysis above, and could occur in the centre or edge of the domains, so a full picture of the size distribution of the domains can only be found by removing them. However, this will mean our diameter analysis will also find the diameters of lithium ions solvated outside of the domain of interest. To allow for comparison, we also showed the calculated diameters found on deleting only water atoms, only anion atoms, and only lithium ions in Fig. S12.

The probability distribution functions for the aqueous domains (Fig. 8(a)) show an initial peak at ∼3.5 Å for each concentration and a shoulder which occurs at ∼5.8 Å at 11.9 mol kg^−1^ and ∼5.4 Å at 19.7 mol kg^−1^.

The first peak arises from single atoms and occurs due to density and packing constraints. It will be made up of contributions from both lithium ions solvated in the non-aqueous domain and from isolated arrangements of water molecules (*e.g.* water molecule chains or water molecules dissolved in the non-aqueous phase). Larger water-rich regions are encapsulated by the shoulder, which is several water molecules and/or lithium ions across at each concentration. There is a slight narrowing of these domains as the concentration is increased, seen in both a shift of the peak and a reduction in the length of the tail at large diameters. However, this is not enough to account for the fall in water molecules observed in water-only domains in the preceding cluster analysis (Fig. 6). Instead, these results suggest that water channels are broken up by anions as the salt concentration is increased, leading to isolated and narrow water domains, as has been seen in previous simulation and experimental studies.^25–28^

The distribution functions for the diameters of the non-aqueous domains (Fig. 8(b)) show two distinct peaks at each concentration: the first at ∼3.3 Å at both concentrations investigated, and the second at ∼6.1 Å at 11.9 mol kg^−1^ and at ∼6.3 Å at 19.7 mol kg^−1^.

Again, the first peak arises due to packing constraints and will include contributions from lithium ions solvated in the aqueous domain as well as from arrangements of NTf_2_ ions that leave bonds to single atoms pointed away from the bulk of the ion. The second peak arises due to domains of multiple ions and occurs at a diameter larger than the breadth of a single ion at each concentration.^53^ This suggests that the anion domains are consistently more than a single anion across at each concentration and points to a true separation between anion and aqueous domains. We observed the expansion of the diameter of these domains as the concentration was increased; however, this expansion of the most likely domain diameter is matched by the contraction in the size of the water domains, which accounts for the consistent domain length scale seen in the experimental data of this and previous studies.^20,23^ The expansion is more notable in the tail of the distribution at large diameters, with diameters of ∼10 Å emerging at 19.7 mol kg^−1^. In total this shows that the anion domains are narrow, between one and two anions across; however, our previous cluster analysis (Fig. 7) has shown that these domains do extend throughout the system at each concentration. This kind of cluster can only arise from narrow but extended anion channels.

In total, this cluster and diameter analysis reveals a picture of an extended anion domain formed of narrow but percolating channels surrounding numerous unconnected narrow water channels. As the concentration is increased, we observed a decrease in the number of water molecules in pure water clusters and a small decrease in the breadth of the water channels. We observed an extended anion network at both concentrations, and we observed slight growth in the probability of broader anion channels as the concentration was increased; however, we observed that the total correlation length scale of the domain structure is maintained as the concentration is increased. Our results show that there is no percolating aqueous domain, and therefore, lithium ions cannot diffuse solely through the aqueous regions. Instead, there must be some diffusion through the anion domain, although whether this takes place *via* a vehicular mechanism in which the anion domains move to link aqueous regions allowing diffusing lithium to remain solvated primarily by water in a vehicular type mechanism, or whether lithium ions in the anion domain also contribute significantly to ion transport will require further work to disentangle. The EPSR method (and structure inversion methods in general^54^) is not well-suited to determining transport properties, so we therefore propose that future computational studies that emphasise transport properties should ensure structural consistency with this work to further validate their findings.

## 4. Conclusions

In this work, we studied the archetypal water-in-salt electrolyte LiNTf_2_ at two concentrations using neutron total scattering. The scattering reveals a small *Q* peak that suggests the presence of a nanostructure with a characteristic length scale of approximately 1.2 nm at each concentration. We used empirical potential structure refinement techniques, refining against multiple different water scattering contrasts, to build a simulation box that matches the experimental scattering profile, and analysed this box to study the emerging solvation structure and nanostructure. Solvation of the anion in particular reveals anion clusters bound by hydrophobic associations, with anion geometry changing as water is removed from the system. We also observed significant disruption to the water solvation structure as the salt concentration was increased, highlighting the deviations from behaviour seen in dilute aqueous electrolytes. Cluster analysis shows that the salt solutions are separated into extended anion-dominated domains that percolate through the whole system, with smaller aqueous domains that appear to take the form of narrow channels. These water channels can contain many molecules but never occur throughout the entire system, suggesting a picture of broken-up aqueous regions. Lithium ions are solvated in both domains, and each domain will have a critical role to play in the transport of ions through the electrolyte. We therefore propose that future computational studies should prioritise transport mechanisms consistent with the structural features reported in this work.

More broadly, this study provides new insights into the solvation and nanostructure present in water-in-salt electrolytes. These results contribute to the molecular understanding of concentrated electrolyte structures, which is critical in the design and manufacture of next-generation, green and environmentally friendly energy storage devices.

## Author contributions

T. S. G. and J. E. H. conceived the research, prepared the samples, performed the experiments, analysed the data and prepared the manuscript. K. J. A. prepared the samples and performed the measurements. S. M. analysed the data. S. P. conceived the research and prepared the manuscript. T. F. H. conceived the research, performed the experiments and analysed the data. T. G. A. Y. analysed the data. G. N. S. conceived the research. All authors contributed to the discussion and writing of the manuscript.

## Conflicts of interest

There are no conflicts to declare.

## Supplementary Material

EB-001-D5EB00105F-s001

## Data Availability

Raw neutron scattering data for this article are available in the ISIS Data Catalogue (https://doi.org/10.5286/ISIS.E.RB2210023-1). Dissolve simulations and GudrunN files are available from the Oxford University Research Archive (https://dx.doi.org/10.5287/ora-gwjevek2j). Supplementary information, including synthesis of ^7^LiNTf_2_, simulation force field parameters, densities of LiNTf_2_ solutions as a function of concentration, schematics of analysis methods used in the main text, total structure factors and total pair distribution functions with their respective fits, comparison to literature structure factors, coordination number histograms, additional spatial density functions, C–C intramolecular distance distributions, running coordination numbers and domain diameter distributions is available. See DOI: https://doi.org/10.1039/d5eb00105f.
