# Supplementary material for: Lithium solvation and anion-dominated domain structure in water-in-salt electrolytes
Source: EES Batter. 2025 Aug 6;1(6):1797–808. doi: 10.1039/d5eb00105f (PMC12372462; doi:10.1039/d5eb00105f)
Supplement: EB-001-D5EB00105F-s001 [file EB-001-D5EB00105F-s001.pdf]

Supplementary Information (SI) for EES Batteries.
This journal is © The Royal Society of Chemistry 2025

Supplementary Information

Lithium solvation and anion-dominated domain structure in water-in-salt electrolytes

Timothy S. Groves*[a], Kieran J. Agg[a], Shurui Miao[a], Thomas F. Headen[b], Tristan G. A. Youngs[b], Gregory N. Smith[b], Susan Perkin[a] and James E. Hallett*[c]

[a] *Physical and Theoretical Chemistry Laboratory, Department of Chemistry, University of Oxford, Oxford OX1 3QZ, UK. E-mail: timothy.groves@chem.ox.ac.uk.*
[b] *ISIS Neutron and Muon Source, Rutherford Appleton Laboratory, Didcot OX11 0QX, UK.*
[c] *Department of Chemistry, School of Chemistry, Food and Pharmacy, University of Reading, Reading RG6 6AD, UK. E-mail: j.e.hallett@reading.ac.uk.*

1 Experimental

1.1 *Synthesis of ^{7}LiNTf$_2$*

A sample of enriched $^7LiNTf_2$ was prepared following the method described by Maeda *et al.*[1] We dissolved 20.56 g of $HNTf_2$ (Fluorochem, 98 %) in $\sim$ 80 cm^3 water (MilliQ, 18.2 MΩ cm^{-1}, 3.0 ppb TOC). 2.69 g of$^7Li_2CO_3$ (Merck, 99 atom%) was added gradually while stirring at room temperature. The resulting solution was stirred for $\sim$ 3 hours before being filtered and dried under vacuum for 3 days at 75°C, resulting in a white crystal.

1.2 *Force Field Parameters*

The force fields used in this work are taken from the literature as Lennard-Jones parameters, with bond and angle parameters. The water force field is based on the flexible simple point charge water (SPC/Fw) model[2], the NTf_2 anion force field is based on that first suggested by Koddermann *et al.* and refined by Neumann *et al.*[3,4], and the lithium force field is taken from a work by pethes *et al.*[5] These are presented in tables S1, S2, and S3.

Table S1. Lennard-Jones parameters σ and ϵ, and charges q, for all atoms in this study.

Atom type		σ / Å	ϵ / kJ mol^{-1}	q / e
Water	O	3.165	0.650	-0.820
	H	0	0	+0.410
Anion	N	3.250	0.213	-0.690
	S	4.083	0.314	+1.076
	O	3.463	0.264	-0.579
	C	3.150	0.083	+0.494
	F	2.655	0.067	-0.189
Cation	Li	2.126	0.076	+1.000

Table S2. Equilibrium bond lengths r_{eq} and force constants k in this study.

Bond type		r_{eq} / Å	k / kJ mol^{-1} Å^{-2}
Water	H-O	0.960	4431
Anion	N-S	1.570	3113
	S-O	1.442	5331
	S-C	1.818	1979
	C-F	1.323	3697

Where necessary, additional repulsive interactions were added to specific pair potentials to act as a hard wall and prevent the formation of unrealistically close associations in the empirical potential. The affected

Table S3. Equilibrium bond angles and force constants k in this study.

Angle type		Equilibrium bond angle / °	k / kJ mol^{-1} rad^{-2}
Water	H-O-H	107.1	318
	S-N-S	125.6	671
	N-S-O	113.6	789
	C-S-N	100.2	816
Anion	O-S-O	118.5	969
	C-S-O	102.6	870
	F-C-S	111.8	694
	C-C-F	107.1	781

pair potentials were the $F_{anion}-F_{anion}$ and $H_{water}-F_{anion}$, where contacts could be within the fluorine atom Lennard-Jones radius, and $H_{water}-H_{water}$ interactions, where contacts for a small fraction of particles were significantly shorter than the expected hydrogen bond length in pure water[6]. The additional potential took the form of a Guassian distribution and was centred at the 0 position of the pair potential. These additional Gaussian potential terms mimicked the steric repulsion between atoms and are given in table S4. No deviation from the structure of pure water was found after refining a Dissolve simulation box using the new $H_{water}-H_{water}$ potential against experimental data[6].

Table S4. Additional Gaussian potentials added to specific pair potentials. In each case, the additional potential is centred at x=0 and defined by a magnitude A and a full-width-half-maximum.

Pair potential	A / kJ mol^{-1}	$FWHM$ / Å
F_{anion}-F_{anion}	1×10^9	0.95
H_{water}-F_{anion}	1×10^7	0.77
H_{water}-H_{water}	5×10^7	0.77

1.3 Densities

The densities of aqueous $LiNTf_2$ samples were measured using a density meter (Anton Paar DMA 4100 M). Briefly, a small amount of sample ($\sim$ 1 cm^3) was injected into a U-shaped capillary tube which is oscillated. The frequency of the oscillation is measured and can be used to determine the density of the sample. Densities were measured at 25°C in the concentration range 3.1 - 19.8 mol kg^{-1} and are shown in figure S1.

1.4 Cluster and Void Analyses

As described in the main text, we study extended structures within the simulated trajectory using two methods: a cluster analysis to determine the number of molecules present in extended clusters, and a void

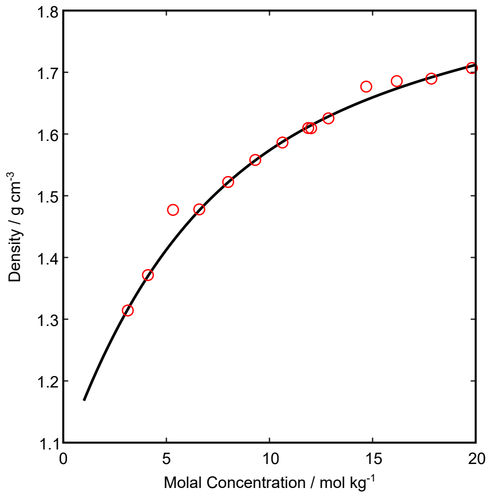

Figure S1. Density of aqueous $LiNTf_2$ solutions as a function of concentration. Experimental points are shown as open red circles. The black line shows the trend in the data.

analysis to determine the dimensions of clusters. These analyses are briefly described below and are shown schematically in figures S2 and S3.

Cluster analysis, figure S2: A specific interaction of interest is chosen, and a cutoff for that interaction, defined as the minimum after the first peak in the $g(r)$, is selected. For example, when studying water-only clusters, we look at the hydrogen bonding interaction, defined by an intermolecular O-H distance of < 2.3 Å. We then search through the trajectory file for a molecule involved in the chosen interaction, find the atom defining the interaction, and then search the trajectory file for the corresponding atoms on a different molecule within the selected cutoff. Any molecules within this cutoff are labelled as belonging to the same cluster as our initial molecule. This is then repeated for each of those molecules until no new molecules are detected in that cluster. We then move on to a different molecule that is not present in the first cluster and begin again. All molecules of interest for each frame in the trajectory file are studied to build up a full picture of clustering in the system.

Void analysis, figure S3: For each area of interest, selected atoms are deleted, leaving empty 'voids'. For example, when studying molecules in the aqueous domain, all atoms in water molecules and lithium ions are deleted, leaving behind an aqueous domain void. A random point b at a minimum of 1.2 Å from any remaining atoms is chosen. The two nearest neighbouring atoms a and c are then found and the angle abc is calculated. If the angle $abc > 3$ radians (*i.e.* is close to 180°) then atoms a and c are on opposite sides of the domain and the distance ac provides an estimate of the domain diameter.

In our analysis, a trajectory of 10000 frames is studied every fifth frame, meaning 2000 frames are studied in total for both the cluster and void analyses. For the void analysis, within each frame 100,000 points b are chosen to ensure reliable statistics are obtained.

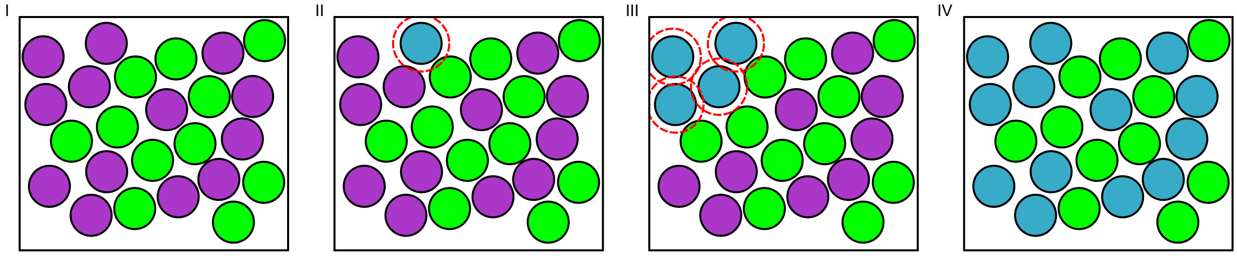

Figure S2. Schematic showing the process in the cluster analysis. *I* shows a frame for a trajectory containing two types of molecule, A (purple) and B (green). To study clusters of molecules of type A, an A molecule is selected, as highlighted in *II*, and the vicinity of that molecule is searched for further A molecules. In this case, only one other A molecule is found within the defined interaction cutoff, and is labelled as belonging to the same cluster as the first. The vicinity of that second A molecule is then searched and again one new A molecule is found. This process is repeated until no new A molecules are found within the cutoff distance of any A molecules already in the cluster. When this occurs, the cluster is finished, in this case containing four A molecules, as shown in *III*. The process is then repeated for further A molecules in the trajectory until all molecules of interest belong to a single cluster. *IV* shows that in this example frame, there are four distinct clusters of A molecules containing one, three, four and five molecules. The process is then repeated for the next frame in the trajectory file.

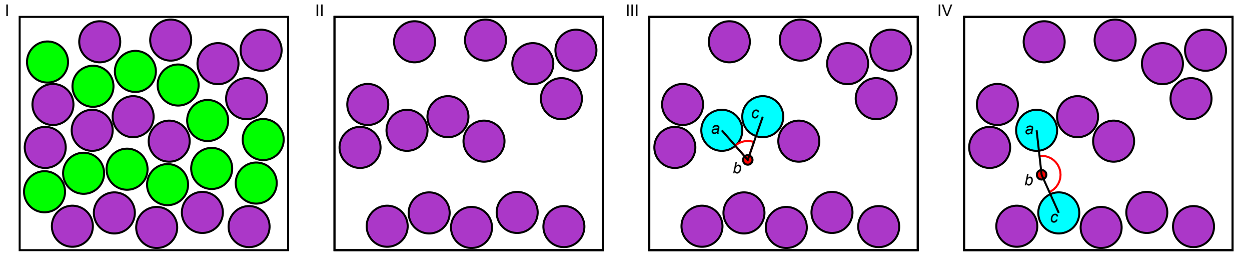

Figure S3. Schematic showing the process in the void analysis. *I* shows a frame for a trajectory containing two types of molecule, A (purple) and B (green). To study clusters of molecules of type B, all B molecules are deleted, as shown in *II*. A point b is then selected, outside of any remaining atoms, and the two closest atoms a and c are found. *III* and *IV* show two example selections of b. In *III*, the angle abc is smaller than 3 radians so the distance ac does not correspond to a diameter of the B clusters and the measurement is discarded. In *IV*, the angle abc is greater than 3 radians because atoms a and c are on opposite walls of the void left behind on deleting the B molecules. This means the distance ac corresponds to a measurement of the diameter of the B molecule clusters.

2 Results and discussion

2.1 Experimental fits

Comparisons of the measured and fitted total structure factors, $F(Q)$s, and the real space total pair distribution functions, $G(r)s$, are shown in figure S4 at 11.9 mol kg^{-1} and figure S5 at 19.7 mol kg^{-1}.

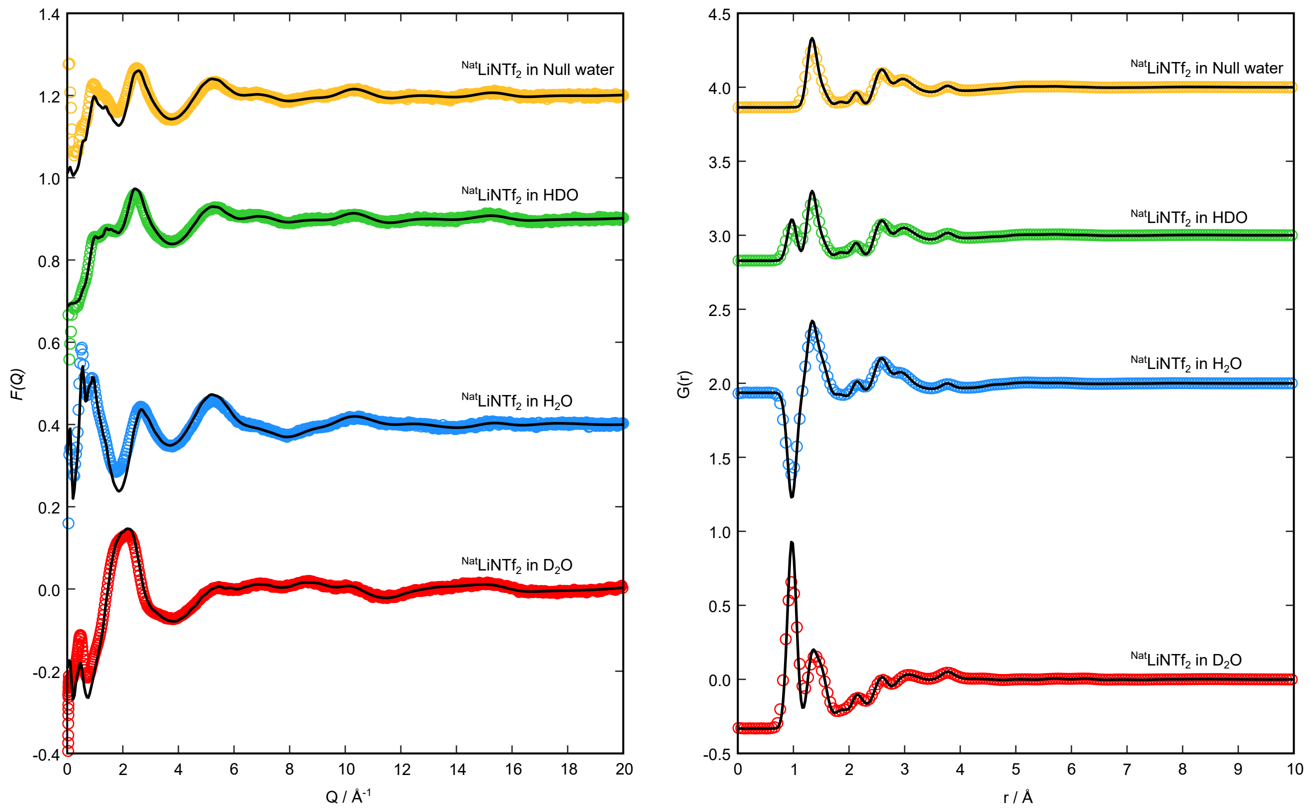

Figure S4. Total structure factors, $F(Q)$ (*left*), and total pair distribution functions, $G(r)$ (*right*) for different solvent contrasts of aqueous $LiNTf_2$ at 11.9 mol kg^{-1}. Experimental data points are shown as open circles and the Dissolve model fit data is shown by the solid black lines.

For the $F(Q)$s, in each case there is excellent agreement between the experimental data and the fit generated from the Dissolve simulations for all $Q > 0.2$ Å^{-1}, suggesting the simulated boxes provide a good model for the behaviour of aqueous $LiNTf_2$ solutions at each concentration. There is a slight baseline mismatch in some cases at 0.2 Å^{-1} $< Q <$ 1.0 Å^{-1} and agreement is quantitative at $Q > 1.0$ Å^{-1}. Discrepancies likely arise due to a poor inelasticity correction as a result of H_2O contamination. However, the fitted scattering is obtained from a best fit across all contrasts, so discrepancies in some contrasts should not affect the interpretation significantly. Agreement is also excellent between the experimental $G(r)$s and the simulated fit. A zoomed-in image of the $F(Q)$s for $^{Nat}LiNTf_2$ in H_2O and D_2O is shown in the main text figure 1.

Previous characterisation of these systems has taken place using small angle neutron scattering (SANS), neutron diffraction, small angle X-ray scattering (SAXS), and X-ray total scattering measurements, with many previous scattering experiments also performing in depth molecular dynamics simulations to accompany their data. To allow comparison between our data and previous characterisation, we present our

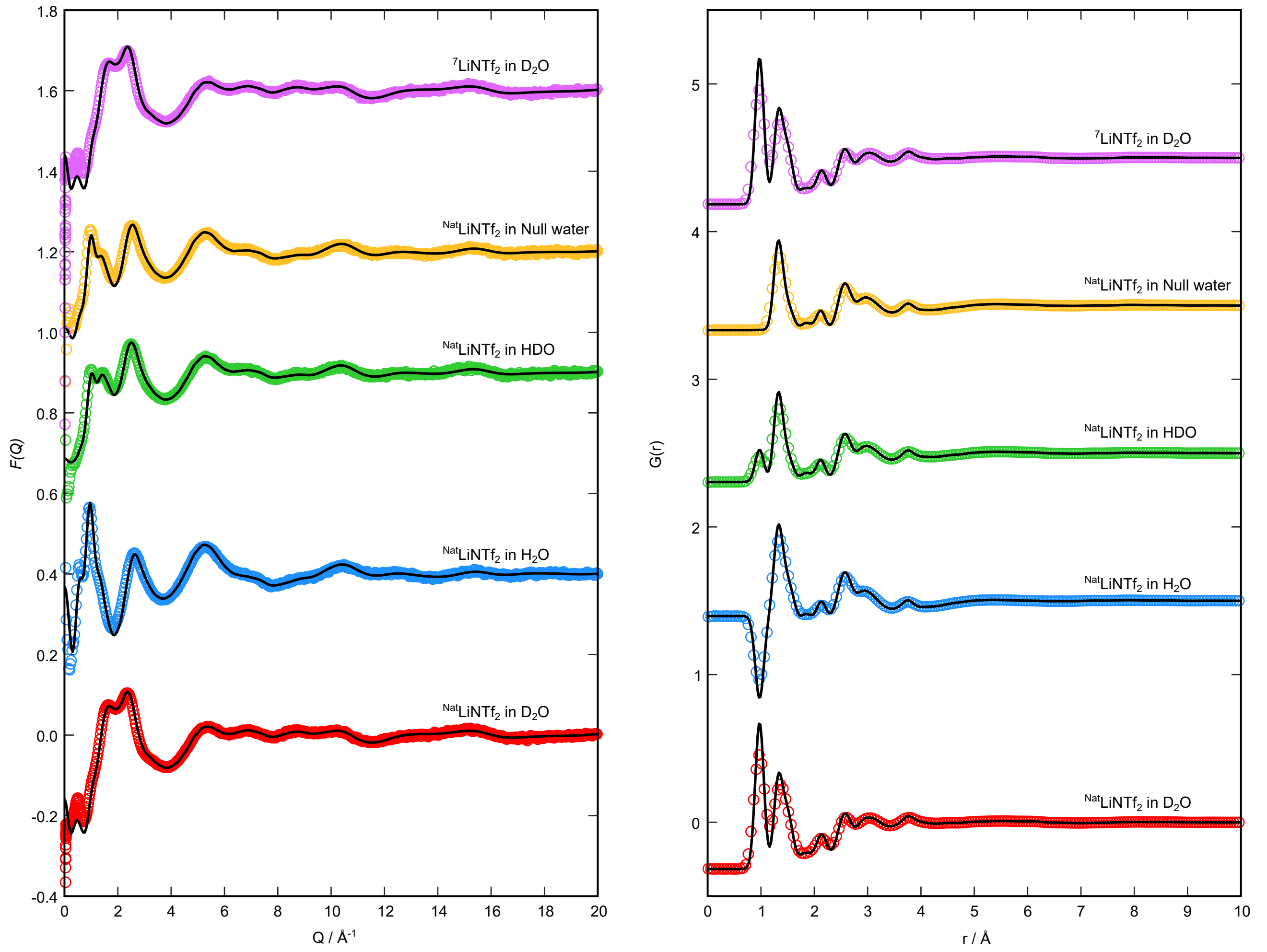

Figure S5. Total structure factors, *F(Q)* (*left*), and total pair distribution functions, *G(r)* (*right*) for different solvent and lithium contrasts of aqueous $LiNTf_2$ at 19.7 mol kg^{-1}. Experimental data points are shown as open circles and the Dissolve model fit data is shown by the solid black lines.

neutron data and simulations of 19.7 mol kg^{-1} $LiNTf_2$ in D_2O alongside literature SANS data and simulations in figure S6. We are also able to generate an X-ray $F(Q)$ from our configurations, which is shown alongside literature SAXS and total X-ray scattering data and corresponding simulations in figure S7.

In general we see no significant difference between the experimentally generated scattering patterns. There are some differences in the simulated scattering, which arise because, unlike in previous cases, our simulation box is refined towards the experimentally measured neutron data. We also note excellent agreement between the experimental X-ray total scattering of Zhang *et al.*[10] and González *et al.*[7], and the X-ray $F(Q)$ generated from our configurations, without any measures taken to refine towards the X-ray data. This means our simulations can capture features of the experimental data that are not seen in pure molecular dynamics simulations.

2.2 Solvation and Nanostructure

Coordination histograms showing the probability of finding a lithium ion with n oxygen atoms inside the first hydration shell (defined as 3.1 Å from the lithium ion centre from the primary *g(r)* minimum shown in figure 3 of the main text), are shown in figure S8. At 11.9 mol kg^{-1} there is a clear preference for near total

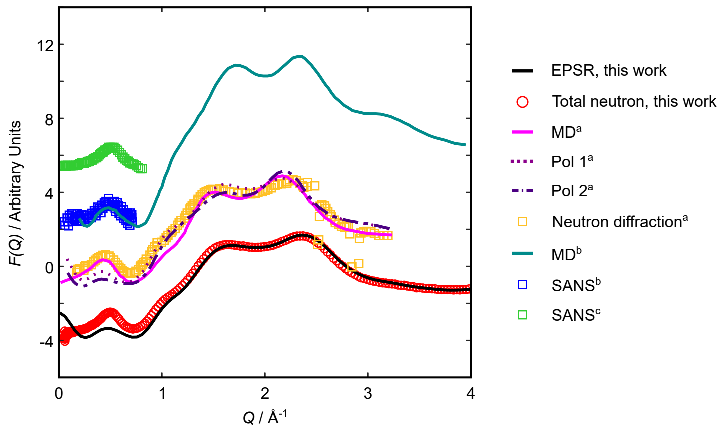

Figure S6. Literature neutron scattering data comparison for ≈ 20 mol kg^{-1} $LiNTf_2$ in D_2O. Total neutron scattering data from this work is shown as red circles, with the simulated $F(Q)$ from the EPSR model shown as a solid black line. Neutron diffraction data from González *et al.*[7], a, is shown as the yellow squares with MD simulations using a non-polarizable (MD, solid pink line) and two polarizable (Pol1, purple dotted line, and Pol2, indigo dashed dotted line) force fields shown alongside. SANS data from Borodin *et al.*[8], b, is shown as blue squares while MD simulations based on the SANS data is shown as the solid teal line. SANS data from Horwitz *et al.*[9], c, is shown as green squares. Data is normalised by the low Q peak at ≈ 0.5 Å^{-1} and are offset to provide clarity.

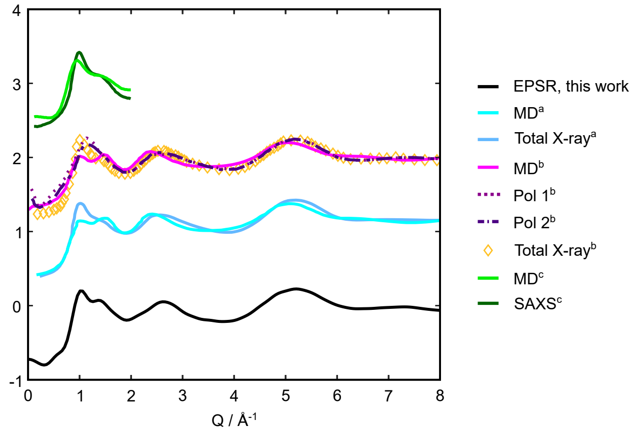

Figure S7. Literature X-ray scattering data comparison for ≈ 20 mol kg^{-1} $LiNTf_2$ in H_2O. A simulated X-ray total scattering $F(Q)$ generated from the EPSR model shown as a solid black line. X-ray total scattering data from Zhang *et al.*[10], a, is shown as the blue line while MD simulations based on the data is shown as the solid cyan line. X-ray total scattering data from González *et al.*[7], b is shown as the yellow diamonds with MD simulations using a non-polarizable (MD, solid pink line) and two polarizable (Pol1, purple dotted line, and Pol2, indigo dashed dotted line) force fields shown alongside. Data from SAXS measurements from Yu *et al.*[11], c, is shown by the dark green line, with MD simulations based on the data shown by the pale green line. Data is normalised by the first Q peak at ≈ 1.4 Å^{-1} and are offset to provide clarity.

solvation of lithium by water molecules, however as the concentration of salt is increased it becomes more likely to find lithium ions solvated by both water molecules and NTf_2 ions.

Spatial density functions, SDFs, showing probability for coordination in the first solvation shell around molecules and groups at 11.9 mol kg^{-1} are shown in figure S9. The first solvation shell is defined as extending to the first minimum in the respective *g(r)*, as shown in figure 3 of the main text. Analagous SDFs at 19.7 mol kg^{-1} are shown in figure 4 of the main text.

The intramolecular C-C bond distance distribution in NTf_2 ions, comparing those coordinated to at least

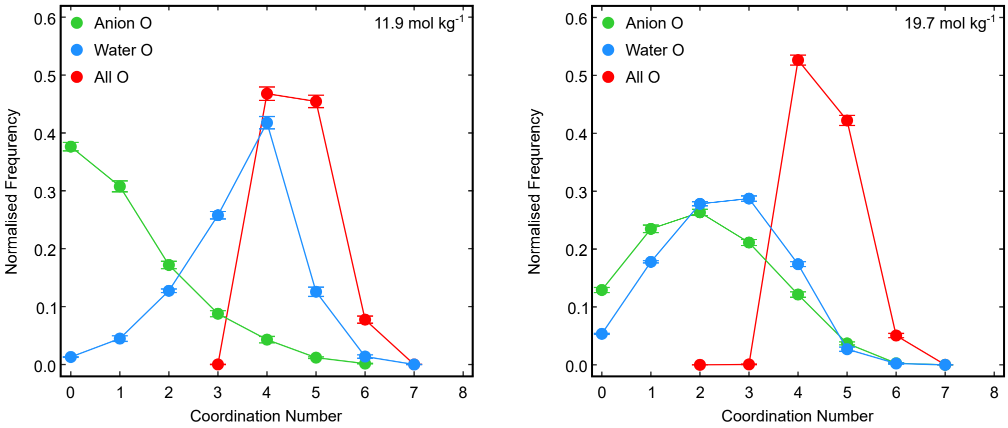

Figure S8. Coordination histograms showing the normalised frequency of finding a lithium ion with n oxygen atoms within 3.1 Å. Histograms are shown for oxygen atoms on the anion (green), water oxygens (blue), and all oxygens (red). *Left:* Coordination histograms at 11.9 mol kg^{-1}. *Right:* Coordination histograms at 19.7 mol kg^{-1}. The lines are drawn as guides to the eye.

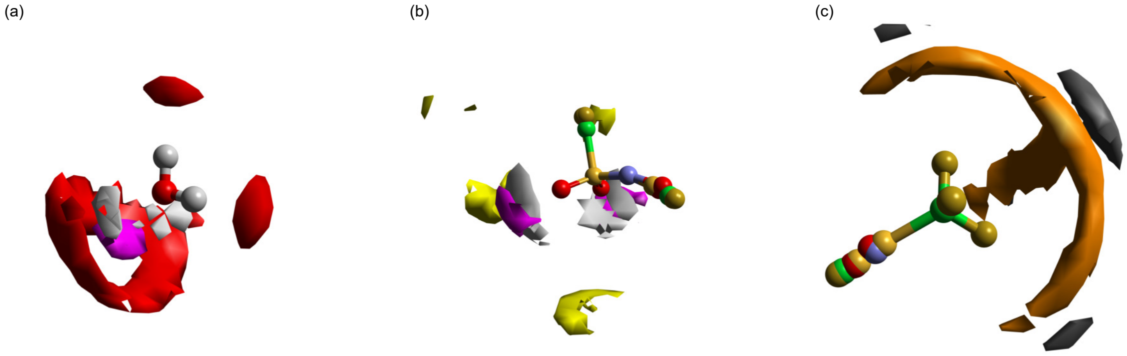

Figure S9. Spatial density functions (SDFs) showing probability surfaces in the first solvation shell at 11.9 mol kg^{-1}. (a): Coordination of water hydrogen (white, 40 % surface), water oxygen (red, 15 % surface), and lithium (magenta, 36 % surface) around a water molecule. (b): Coordination of NTf_2 sulfur (yellow, 15 % surface), lithium (magenta, 10 % surface) and water hydrogen (gray, 30 % surface) around the anion SO_2 group. (c): Coordination of NTf_2 fluorine (orange, 10 % surface) and NTf_2 carbon (black, 2.5 % surface) around the anion CF_3 group.

one lithium ion to those not coordinating any lithium ions, is shown in figure S10. Coordination is defined by the primary minimum in the Li-O $g(r)$ at 3.1 Å.

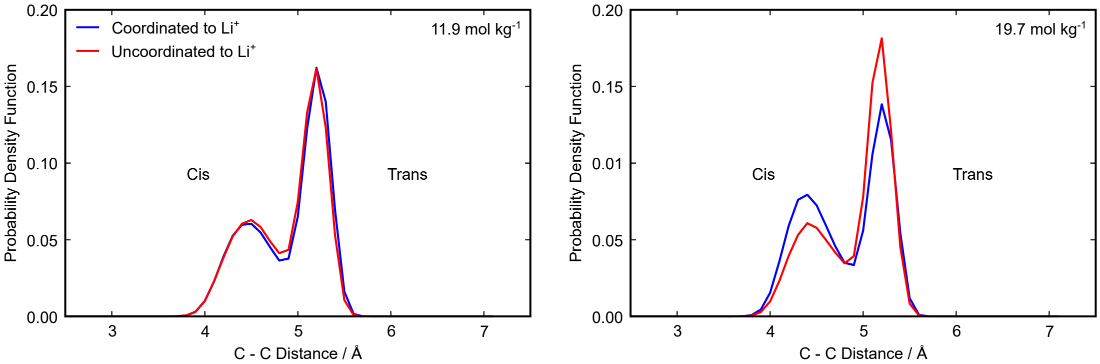

Figure S10. Probability density functions for the carbon carbon distance in NTf_2 ions in the Dissolve simulated box, looking at those coordinated to (blue) or not coordinated to (red) lithium ions. *Left:* the distribution found at 11.9 mol kg^{-1}, where the ratio of coordinated to uncoordinated NTf_2 ions was found to be ≈ 2.4. *Right:* the distribution found at 19.7 mol kg^{-1}, where the ratio of coordinated to uncoordinated NTf_2 ions was found to be ≈ 11.6.

Running coordination numbers showing the change in O_{water}-O_{water} coordination as the distance r is increased are shown in figure S11 for pure water[6] and for the WiS electrolyte at each studied concentration. Cutoffs corresponding to the minimum after the first peak in pure water (3.4 Å) and to the minimum after the first peak in the WiS electrolytes (4.0 Å) are also shown. At all separations, there is greater O-O coordination in pure water, with coordination number decreasing as the concentration of $LiNTf_2$ is increased.

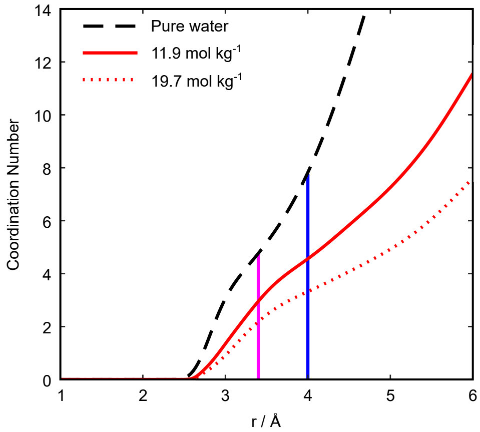

Figure S11. Running coordination number as a function of distance for the O_{water}-O_{water} interaction in pure water (dashed black line), and for the 11.9 mol kg^{-1} (solid red line) and 19.7 mol kg^{-1} (dotted red line) aqueous electrolyte. The cutoff defined by the primary minimum in the pure water $g(r)$ at 3.4 Å corresponding to the O-H hydrogen bonding distance in pure water is shown by the vertical magenta line, while the cutoff defined by the primary minimum in the WiS electrolyte $g(r)$s at 4.0 Å is shown by the vertical blue line.

2.3 Network formation

To quantify the size of nanostructured regions present in aqueous $LiNTf_2$ we perform a modified void analysis as described in the main text. For each area of interest, selected atoms are deleted, leaving empty 'voids'. A random point b at a minimum of 1.2 Å from remaining atoms is chosen. The two nearest neighbouring atoms a and c are then found and the angle abc is calculated. If the angle $abc > 3$ radians (*i.e.* is close to 180°) then atoms a and c are on opposite sides of the domain and the distance ac provides an estimate of the domain diameter. In our analysis, a trajectory of 10000 frames is studied every fifth frame, meaning 2000 frames are studied in total. For each frame, 100,000 points b are chosen to ensure reliable statistics are obtained.

Figure 7 of the main text shows diameters for the voids left on deleting all Li and water O and H atoms (the aqueous domains) and the voids left on deleting all Li and anion N, S, O, C, and F atoms (the non-aqueous domains). However, deleting lithium ions in *e.g.* the aqueous domains leaves small lithium ion sized voids in the non-aqueous domains which are also included in the output probability density function. To allow for comparison, figure S12 shows diameters for the voids left on deleting the water atoms only, the anion atoms only, and the lithium atoms only.

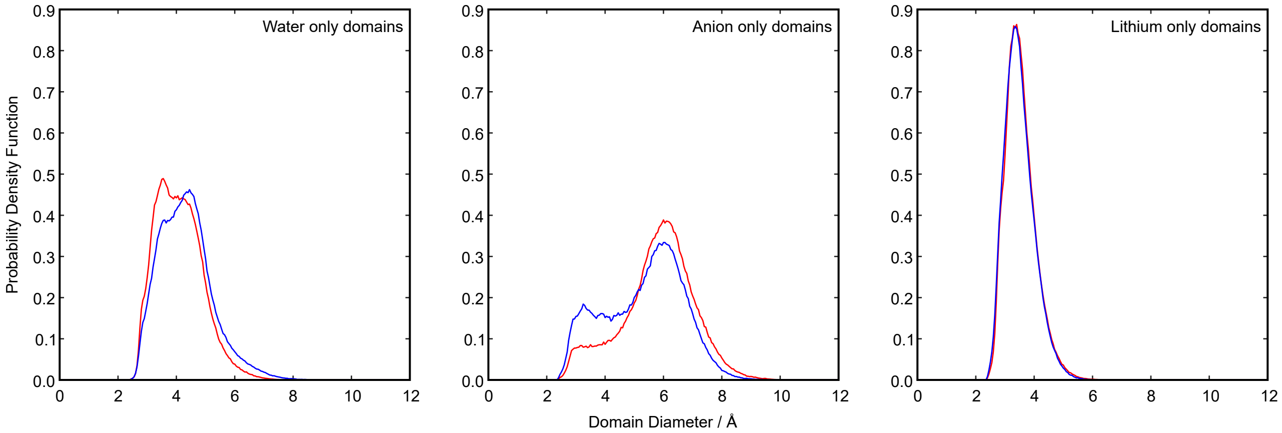

Figure S12. Probability density functions for the diameters of the domains present in concentrated aqueous $LiNTf_2$. Domains found at 11.9 mol kg^{-1} are shown in blue and those found at 19.7 mol kg^{-1} are shown in red. *Left:* Diameters of voids left behind on deleting water molecules only. *Centre:* Diameters of voids left behind on deleting NTf_2 ions only. *Right:* Diameters of voids left behind on deleting Li ions only.

References

[1] S. Maeda, Y. Kameda, Y. Amo, T. Usuki, K. Ikeda, T. Otomo, M. Yanagisawa, S. Seki, N. Arai, H. Watanabe and Y. Umebayashi, *The Journal of Physical Chemistry B*, 2017, **121**, 10979–10987.

[2] Y. Wu, H. L. Tepper and G. A. Voth, *Journal of Chemical Physics*, 2006, **124**, 024503.

[3] T. Köddermann, D. Paschek and R. Ludwig, *ChemPhysChem*, 2007, **8**, 2464–2470.

[4] J. Neumann, B. Golub, L.-M. Odebrecht, R. Ludwig and D. Paschek, *Journal of Chemical Physics*, 2018, **148**, 193828.

[5] I. Pethes, *Journal of Molecular Liquids*, 2017, **242**, 845–858.

[6] A. K. Soper, *International Scholarly Research Notices*, 2013, **2013**, 279463.

[7] M. A. González, H. Akiba, O. Borodin, G. J. Cuello, L. Hennet, S. Kohara, E. J. Maginn, L. Mangin-Thro, O. Yamamuro, Y. Zhang *et al.*, *Physical Chemistry Chemical Physics*, 2022, **24**, 10727–10736.

[8] O. Borodin, L. Suo, M. Gobet, X. Ren, F. Wang, A. Faraone, J. Peng, M. Olguin, M. Schroeder, M. S. Ding *et al.*, *ACS nano*, 2017, **11**, 10462–10471.

[9] G. Horwitz, E. Härk, P. Y. Steinberg, L. P. Cavalcanti, S. Risse and H. R. Corti, *ACS nano*, 2021, **15**, 11564–11572.

[10] Y. Zhang, N. H. Lewis, J. Mars, G. Wan, N. J. Weadock, C. J. Takacs, M. R. Lukatskaya, H.-G. Steinrück, M. F. Toney, A. Tokmakoff *et al.*, *The Journal of Physical Chemistry B*, 2021, **125**, 4501–4513.

[11] Z. Yu, L. A. Curtiss, R. E. Winans, Y. Zhang, T. Li and L. Cheng, *The Journal of Physical Chemistry Letters*, 2020, **11**, 1276–1281.
